# Effects of Physical Exercise on Neuroplasticity and Brain Function: A Systematic Review in Human and Animal Studies

**DOI:** 10.1155/2020/8856621

**Published:** 2020-12-14

**Authors:** Matheus Santos de Sousa Fernandes, Tayrine Figueira Ordônio, Gabriela Carvalho Jurema Santos, Lucas Eduardo R. Santos, Camila Tenório Calazans, Dayane Aparecida Gomes, Tony Meireles Santos

**Affiliations:** ^1^Neuropsquiatry and Behavior Science Postgraduate Program, Federal University of Pernambuco, Recife, PE, Brazil; ^2^Laboratório de Imunopatologia Keizo Asami-LIKA, University of Pernambuco, Recife, PE, Brazil; ^3^Academic Center of Vitoria de Santo Antão, Federal University of Pernambuco, PE, Brazil; ^4^School of Physical Education, University of Pernambuco, Recife, PE, Brazil; ^5^Department of Physiology and Pharmacology, Center of Biosciences, Federal University of Pernambuco, Recife, PE, Brazil

## Abstract

**Background:**

Physical exercise (PE) has been associated with increase neuroplasticity, neurotrophic factors, and improvements in brain function.

**Objective:**

To evaluate the effects of different PE protocols on neuroplasticity components and brain function in a human and animal model.

**Methods:**

We conducted a systematic review process from November 2019 to January 2020 of the following databases: PubMed, ScienceDirect, SciELO, LILACS, and Scopus. A keyword combination referring to PE and neuroplasticity was included as part of a more thorough search process. From an initial number of 20,782 original articles, after reading the titles and abstracts, twenty-one original articles were included. Two investigators evaluated the abstract, the data of the study, the design, the sample size, the participant characteristics, and the PE protocol.

**Results:**

PE increases neuroplasticity via neurotrophic factors (BDNF, GDNF, and NGF) and receptor (TrkB and P75NTR) production providing improvements in neuroplasticity, and cognitive function (learning and memory) in human and animal models.

**Conclusion:**

PE was effective for increasing the production of neurotrophic factors, cell growth, and proliferation, as well as for improving brain functionality.

## 1. Introduction

Environmental stimuli throughout life can result in structural and functional changes in organs and tissues (Westneat et al. [1]). These changes are more propitious in structure that has plastic characteristics, as for example the brain, susceptible to changes from development to aging (Martínez-Morga et al. [2]). This process is called neuroplasticity and is defined as the capacity of the central nervous system to promote the neurogenesis and connections due to psychophysiological and environmental factors (Gulyaeva [3]). In this sense, neuroplasticity occurs with an increase in the production of neurotrophins that generate changes in the growth and differentiation of cell signaling (Kempermann et al. [4]).

Neurotrophins are a family of proteins closely related to the survival, development, and functionality of the central and peripheral nervous systems (Kozorovitskiy and Gould [5]; Yamaguchi et al. [6]). The main neurotrophins involved in the neuroplasticity process are neurotrophic factor derived from the glial cell line (GDNF), nerve growth factor (NGF), neurotrophin 3 (NT3), neurotrophin 4 (NT4), and brain-derived neurotrophic factor (BDNF). Studies show that reduced levels of these neurotrophins, especially BDNF, are responsible for decreased brain functions, such as memory, concentration, and learning (Bekinschtein et al. [7]; Parrini et al. [8]).

The activation process of the BDNF signaling pathway is mainly regulated by tropomyosin-related receptor kinase B (TrkB). A previous study showed that increased expression of TrkB was able to reduce the appearance of brain changes, such as depression (Zborowski et al. [9]). It has also been observed that the activation of TrkB affects neuronal dendritic afforestation, spinogenesis, dendritic growth, and spinal morphogenesis (Guo et al. [10]). Faced with such mechanisms responsible for neuroplasticity, we seek to understand which environmental factors can act to modulate this process.

In this context, PE has been described as an efficient modulator of the health status through increased mitochondrial bioenergetics, adenosine triphosphate (ATP) synthesis, and reduced lipogenesis, reactive oxygen species (ROS) production, endoplasmatic reticulum stress, and proinflammatory cytokine production such as tumor necrosis factor alpha (TNF-*α*) (Broxterman et al. [11]; Nakandakari et al. [12]; Presby et al. [13]; Daou [14]; Tofas et al. [15]). In addition, recent works have shown that PE is able to promote neuroprotection (Byun and Kang [16]; Martland et al. [17]). A study conducted with elderly women found that 12 weeks of aerobic and resistance exercise improved cognitive function and BNDF expression (Byun and Kang [16]). This is due to the unique capability of the skeletal muscle to increase activation to the cellular signaling pathways connected to crosstalk between muscle and brain (Muchlinski et al. [18]; Kato et al. [19]).

The brain is characterized by having a high plastic capacity; it is necessary to elucidate how environmental factors, such as PE, can influence the production of neurotrophic factors providing improvements in brain functionality, through signaling, growth, and cell differentiation. The main aim of this systematic review is to evaluate the effects of different PE protocols on neuroplasticity components in a human and animal study. The second aim is to evaluate the effects of PE on the production of neurotrophic factors, signaling, cell growth and differentiation, and functional outcomes.

## 2. Material and Methods

The present study was performed following the guideline of the PRISMA statement (Moher et al. [20]).

### 2.1. Strategy Search

The researchers searched the scientific literature from November 2019 to January 2020, using the following databases: PubMed (244), ScienceDirect (11,860), SciELO (2), LILACS (96), and Scopus (104). The following search terms were selected using Medical Subject Headings (MESH): (“Physical Exercise” OR “Exercise, Physical” OR “Exercises, Physical” OR “Physical Exercises” AND “Plasticity, Neuronal” OR “Neuronal Plasticities” OR “Plasticities, Neuronal” OR “Neuroplasticity” OR “Neuroplasticities” OR “Neural Plasticity” OR “Neural Plasticities” OR “Plasticities, Neural” OR “Plasticity, Neural”). Reference lists of all included studies were also reviewed for potentially eligible articles.

### 2.2. Study Selection

Two independent reviewers (MSSF) and (GCJS) selected the articles according to the following inclusion criteria: (1) written in English, (2) between the years 2010 and 2019, involving studies, (3) utilizing “physical exercise” or its variations as intervention, (4) different brain tissues, and (5) neuroplasticity, performed in animal and human studies. Articles were included if they fulfilled the following PICOS criteria (*P*articipants: animals and humans, *I*nterventions: physical exercise, *C*omparisons: *PE vs. No PE groups*, *O*utcomes: neuroplasticity components, *S*tudy: animal models and human studies) (Yensen [21]). In the next stage, a comparison was made between searches and evaluation of titles and abstracts according to the eligibility criteria. The selected abstracts were submitted to the second stage of analysis, in which two other independent researchers reviewed the articles completely and, by consensus, excluded articles that did not meet the criteria. The data regarding the characteristics of the samples, methodology, and main outcomes found were extracted from the selected articles.

### 2.3. Data Extract

The reviewers (MSSF) and (GCJS) extracted the data studies on a preestablished database. The data extracted from each study included: (1) study design, (2) sample characteristics, (3) physical exercise intervention, and (4) effects on the neuroplasticity of different brain tissues. All extracted data was entered into a spreadsheet in Excel by the primary researcher and verified by another researcher. Discrepancies were resolved by consensus.

### 2.4. Risk of Bias Assessment

Two independent authors performed an analysis of the risk of bias in the selected studies. The methodological judgment provided by the Revman 5.3.0 program of the Cochrane Handbook program was used (Higgins et al. [22]). The following are among the criteria of the structure of the bias assessment: (1) generation of random sequence, (2) concealment of allocation, (3) masking of participants and personnel, (4) masking of the result evaluation, (5) result data incomplete, (6) selective reporting, and (7) other bias. The studies were classified as low, medium, or high risk of bias.

## 3. Results

### 3.1. Study Selection

**Figure 1:**
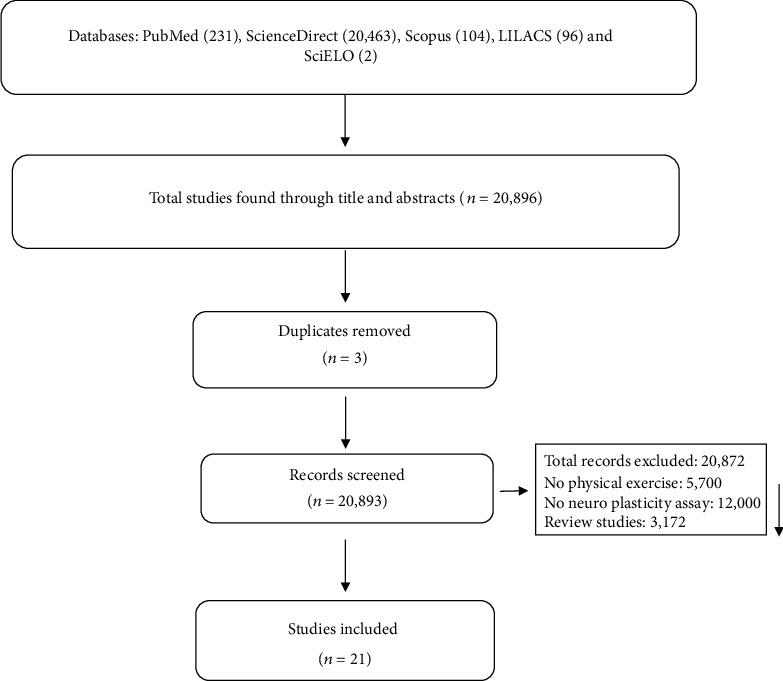
Flow diagram of the studies selected for systematic review.

The flowchart in (Figure 1) shows the successive steps taken to select studies in this systematic review. A total of 12,306 titles and abstracts were selected initially; 11,414 were excluded because they did not comply with the eligibility criteria or were duplicated. Tables 1 and 2 provide the information of the included articles.

### 3.2. Risk of Bias

**Figure 2:**
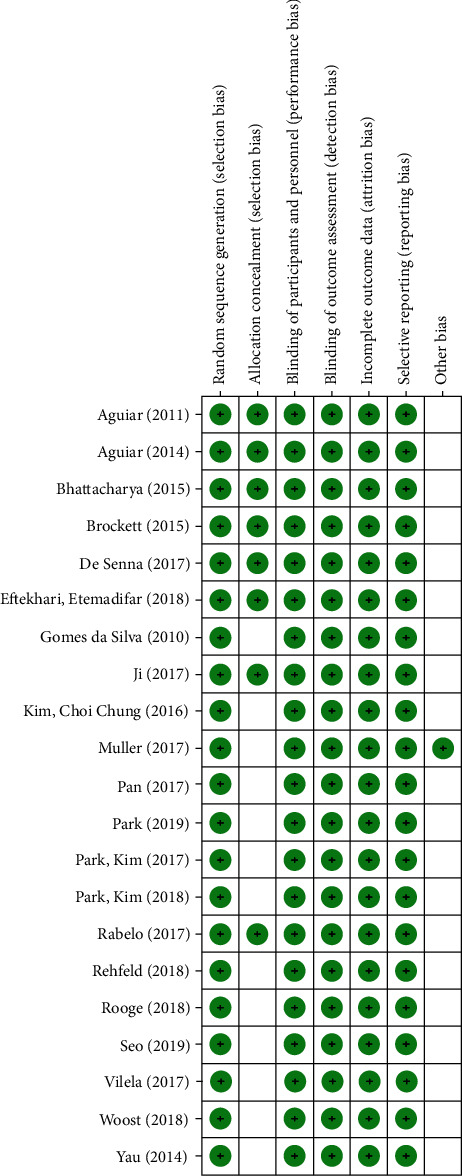
Risk of bias graph: review authors' judgments about each risk of bias item presented as qualitative analysis across all included studies.

**Figure 3:**
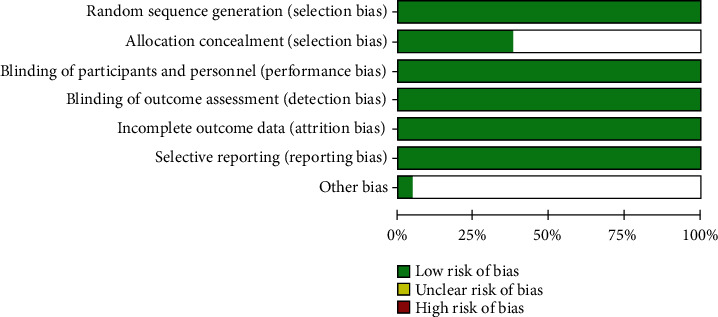
Risk of bias graph: review authors' judgments about each risk of bias item presented as percentages across all included studies.

After this critical evaluation, the twenty-one included studies were classified with low risk of bias (Figures 2 and 3).

### 3.3. Description of Included Studies

We identified 12,306 studies in the databases. Next, 11,414 were removed because they had no data on PE (5201), neuroplasticity analysis (4025), and because they were revision studies (2167). In the end, 21 studies were included (Figure 1). Among the selected, 15 articles were conducted on animals and six in humans.

In animal studies, only one used female exclusively; 13 studies used male animals and one study used both female and male animals. Among eleven studies used, three strains of *Rattus norvegicus* including Wistar (six studies), Sprague-Dawley (four studies), and spontaneous hypertensive rats (SHR) (one study) were used. Four studies were performed with C57BL6 (three studies) and BALB/CJ (one study) mice. The animals were also exposed to models of high-fat diet, diabetes, chemo brain, corticosterone administration, transient middle cerebral artery occlusion, posttraumatic stress disorder, and transgenerational effects of PE. Among the brain area evaluated, 12 studies evaluated the hippocampus, and the others evaluated the prefrontal, orbitofrontal, and entorhinal cortex. Fourteen studies used aerobic PE protocol, of which 11 were on a treadmill and three were voluntary exercise on a running wheel. Only one study used the nonaerobic and strength exercise protocol.

In human studies, only one study used a sample composed exclusively of women. The others used both sexes. The participants' ages ranged from 18 to 80 years old. Among the brain areas evaluated, three studies used magnetic resonance imaging (MRI) to analyze the hippocampus; one evaluated the dorsolateral prefrontal cortex, posterior cingulate, precuneus cortex, hand motor area, occipital lobe, and cerebellum. One of the studies did not report the assessed brain area (Eftekhari and Etemadifar [39]). Of the six selected studies, two studies used strength training, two studies performed dance activities, one study utilizes cycling, one used combined exercise (aerobic, balance, weightlifting, and yoga), and one study used balance and relaxation exercises.

### 3.4. Effects of Physical Exercise on Neurotrophic Factors

In some animal studies (Table 3) using aerobic exercise (treadmill and running wheel training, respectively), increased expression of BDNF protein, receptor levels, and mRNA in the hippocampus was observed (Aguiar et al. [24]; Gomes da Silva et al. [23], Aguiar et al. [25]; Kim et al. [29]; Park and Kim [32]; Vilela et al. [30]; Park et al. [34]). Likewise, a study using resistance training showed an increase in BNDF levels after training sessions (Vilela et al. [30]). In studies with humans, the subjects were submitted to different PE protocols, including pilates, dance, and sports that demonstrated elevated serum and plasma BDNF levels that were evaluated by the ELISA method (Müller et al. [40]; Eftekhari and Etemadifar [39]; Rehfeld et al. [42]) (Table 4). However, when different cycling intensity patterns were used, no differences were observed on BNDF serum levels (Woost et al. [43]).

Six works evaluated TrkB receptor expression in animal studies (Table 3). Five studies observed increased TrkB expression in the hippocampus after aerobic training (Gomes da Silva [23]; Kim et al. [29]; Park and Kim [32]; Park et al. [33]; Park et al. [34]). Only one study identified a reduction in TrkB mRNA level when compared to control after exercise protocol intervention (Vilela et al. [30]). Regarding GDNF level, two studies showed that aerobic exercise was able to increase protein and mRNA levels (Rabelo et al. [37]; Vilela et al. [30]). The p75 neurotrophin receptor (p75NTR) was evaluated by Vilela et al. (Vilela et al. [30]) which showed an increase in P75NTR expression in both aerobic and resistance training protocols compared to untrained animals. Aerobic exercise was also able to increase the levels of postsynaptic density protein 95 (PSD-95), phosphorylated N-methyl-D-aspartate receptor (pNMDA), and synapsin (SYN) when compared to the control (Yau et al. [35]; Brockett et al. [28]; Vilela et al. [30]) and hypertensive groups (Pan et al. [36]).

### 3.5. Effects of Physical Exercise on Cellular Signaling Factors That Stimulate Neuroplasticity

The expression of cellular signaling factors was investigated only in animal studies (Table 3). The different aerobic protocols (treadmill and running wheel) increased expression and phosphorylation of alpha serine/threonine kinase (AKT), cyclic adenosine monophosphate-responsive element-binding protein (CREB), and cyclic adenosine monophosphate (cAMP) phosphorylation (Aguiar et al. [24]; Aguiar et al. [25]; Vilela et al. [30]) in the hippocampus. CREB levels were also increased after strength training (Vilela et al. [30]). Sprague-Dawley rats that were treated with corticosterone, a glucocorticoid released by the adrenal cortex, obtained increased levels of insulin-like growth factor-1 (IGF-1) in the hippocampus after performing aerobic and strength training (Yau et al. [35]).

### 3.6. Effects of Physical Exercise in Neuronal Growth and Differentiation

Only protocols with aerobic exercises evaluated cell growth and differentiation of brain areas. Neurogenesis was evaluated in animal studies. In this sense, PE promoted an increase in the number of cells in the dental gyrus, hippocampus, prefrontal cortex, and orbital cortex (Bhattacharya et al. [27]; Brockett et al. [28]; Kim et al. [29]; de Senna et al. [31]; Pan et al. [36]; Park et al. [34]). However, in the perirenal cortex, these responses after PE were not observed (Brockett et al. [28]). In addition, PE increased cell differentiation, proliferation, and survival in the hippocampus of Sprague-Dawley rats (Yau et al. [35]; Park and Kim [32]). PE also increases the density of neuronal fibers and dendritic column in animal studies (Gomes da Silva et al. [23]; Brockett et al. [28]). In humans, MRI was used to assess neural structures (Table 4). An increase in the mass of white and gray matter was observed in different regions (hippocampus, cortex, cerebellum, and basal ganglia) (Ji et al. [41]; Müller et al. [40]; Rehfeld et al. [42]; Rogge et al. [44]).

### 3.7. Effects of Physical Exercise on Cognitive Abilities

In Wistar rats, a reduction in latency (Aguiar et al. [24]; Gomes da Silva et al. [23]; Vilela et al. [30]) and length of the swimming track (Aguiar et al. [25]) were observed through the water maze test and Barnes' maze, in trained Wistar rats with aerobic exercise and of strength compared to control (Table 3). Better spatial memory capacity (Kim et al. [29]) and both short-term (Park et al. [33]) and long-term (Park et al. [34]) were also observed in the Y-maze and water maze tests after the aerobic exercise protocol in Wistar rats and C57BL/6 mice. Wistar rats and C57BL/6 mice trained in aerobic exercise showed faster learning (Aguiar et al. [25]) and increased spatial learning (Aguiar et al. [25]) after the water maze and Morris water tests. When aerobic PE was performed by the parents, an increase in the puppies' spatial learning capacity (Sprague-Dawley rats) was also demonstrated (Park and Kim [32]).

In humans, the tests to assess memory were verbal learning test, attentional performance test, immediate, delayed, and recognition recall from Hopkins Verbal Learning Test-Revised, immediate and delayed story recall from the rivermead behavioral memory test, WAIS-III digit symbol substitution modality test, WAIS-III digit span, trail making test, Stroop Color and Word Test, digit span forward and backward of the Wechsler Memory Scale, Rey-Osterrieth complex figure test, VVM, subtests “figures,” “dices,” and “matrices” from the Intelligence Structure Test 2000R, and “California Verbal Learning Test” (Table 4). In humans, memory improvements have been demonstrated in combined exercise (Ji et al. [41]) and dance and strength training protocols (Rehfeld et al. [42]). After strength training, dancing and cycling improvements in cognition were also observed (Müller et al. [40]; Woost et al. [43]). Both strength training and dance cause better responses about a short-term recall, long-term recall, verbal long-term recognition, and attention reaction (Müller et al. [40]). Woost et al. did not observe differences on cognition and memory after cycling training.

Two studies evaluated the effect of aerobic exercise on anxiety through the elevated plus maze test and open field test in the animal (Table 3). It was observed that the trained animals had increased time of exploration with open arms, locomotion, and time of exploration (Aguiar et al. [25]; Seo et al. [38]). In the open field test, there was an increase in exploration in the central regions, in addition to an increase in locomotion (Aguiar et al. [25]). Depression was evaluated in three studies using the tall suspension test (Seo et al. [38]) and forced swim test (Rogge et al. [44]; Seo et al. [38]) protocols. In all studies, it was observed that trained animals had reduced immobility after exercise compared to the control groups (Yau et al. [35]; Rogge et al. [44]; Seo et al. [38]). In models with corticosterone administration, aerobic exercise was also able to reduce immobility time (Yau et al. [35]). In humans, no study has evaluated anxiety and depression parameters.

## 4. Discussion

The present review confirmed the hypothesis that different modalities of PE (aerobic and resistance training) are capable of potentiating neuroplasticity in different species (animals and humans), through the high production of neurotrophic factors, cell signaling, growth, and development, resulting in improved cognition. In addition, it was also found that PE improved functional abilities such as state of anxiety and depression in animals. PE, especially aerobic exercise, was responsible for the high expression of brain trophic factors. Sleiman et al. [45] observed the increase in BDNF levels in the hippocampus after thirty days of free access to the running wheel in mice. The increase in this neurotrophic factor, according to the authors, may occur due to endogenous production and affinity of *β*-hydroxybutyrate, by BDNF promoter regions (Sleiman et al. [45]).

Park et al. [33] observed that paternal exercise was able to increase BDNF levels in the hippocampus, demonstrating a transgenerational effect like that observed in the study by Yin et al. [46]. In this study, it was demonstrated that paternal exercise provided increased levels of BDNF in the offspring (Yin et al. [46]). The changes promoted by exercise can be transmitted by epigenetic mechanisms, as well as the decrease in DNA methylation in the offspring's hippocampus (Dyer et al. [47]).

In humans, results like those in animal studies have been observed. An increase in serum/plasma BDNF was observed after aerobic (dance and sports) and anaerobic (Pilates) exercise. Hakansson et al. [48] realized a study with healthy voluntaries who performed moderate-intensity aerobic exercise. They observed an increase in BDNF levels in the elderly's bloodstream after thirty-five sessions of aerobic exercise analyzed by ELISA (Hakansson et al. [48]). BDNF produced peripherally can act as a metabolite in response to the contraction of skeletal muscle (Jiménez-Maldonado et al. [49]). This compound is carried by the blood stream crossing the blood-brain barrier, promoting its activation in the central nervous system (Jiménez-Maldonado et al. [49]). When performing a cycling protocol in high-intensity training (HIT), Woost et al. (2018) showed no increase in BDNF levels.

Freitas et al. (2018) used a different HIT treadmill protocol for six weeks in Wistar rats, showing increase in the expression of BDNF in the hippocampus. The increase in BDNF levels occurs due to cell signaling of TrkB, IGF-1, and vascular endothelial growth factor (VEGF) (Hurtado et al. [50]). In addition, the difference between the HIT protocols (frequency, duration, and on the training surface) can generate divergent results (Jiménez-Maldonado et al. [51]). The present review also demonstrated that aerobic exercise was able to increase the expression of PSD-95, pNMDA, and SYN, in healthy and hypertensive models. Ploughman et al. [52] performed a resistance exercise protocol at moderate intensity for sixty minutes/five times a week in an animal model of cerebral ischemia. This demonstrates that PE can promote neuroplasticity even in pathological and adverse conditions of the brain (Ploughman et al. [52]).

PE is responsible for activating transcriptional factors linked to cell metabolism (Egan and Zierath [53]). In this perspective, CREB, AKT, and cAMP had their signaling increased in the hippocampus after PE protocols at different frequencies (12 days, 4 and 6 weeks). Studies demonstrating the expression of these cellular components play a crucial role in central nervous system development and maturation (Pulimood et al. [54]). In this sense, Jung and Kim [55] evaluated the effect of forced training on a treadmill without inclination, for thirty minutes in five weeks in animals with sensorimotor restriction. The results in the brain demonstrate that there was a high stimulation of AKT and phosphatidylinositol 3 kinase (PI3K), being associated with improvement in memory and motor control (Jung and Kim [55]). Therefore, it is suggested that the increase in these factors may be implicated in enhancing neuronal survival, synaptic plasticity, cognitive function, and neurogenesis (Rodríguez-Tornos et al. [56]).

Aerobic and anaerobic exercise was able to increase insulin activity and its transcription factor (IGF-1) expression in animals treated with corticosterone. It has been observed that EF plays a functional role on the activation of IGF-1 (Llorens-Martín et al. [57]). Furthermore, a short intervention with exercise was needed to reduce the stressful response of cortisol administration and associate with IGF-1 (Yau et al. [35]). Nevertheless, the protective effects of PE against physical and mental stress are already consolidated in the scientific literature (Salmon [58]).

Recent studies have observed the association of IGF-1 and CREB with high stimulation of AKT and cellular neuroplasticity and brain functions (Tabei et al. [59]; Caracciolo et al. [60]). Additionally, neuronal growth and differentiation may happen in response to environmental stimuli including PE (Ma et al. [61]). On the other hand, changes in cell number and neuronal density were observed in animal models, as well as an increase in gray and white matter in human models. Nevertheless, Tabei et al. [59] observed the isolated effect of PE (40 sessions for 1 year) on white and gray matter through magnetic resonance imaging in the elderly. The authors found no difference in brain volume after an isolated PE protocol (Tabei et al. [59]). Inherent in this, the aging process promotes a natural reduction in brain mass; this may be the explanation of why exercise did not provide significant changes in these components (Zhang et al. [62]).

Both types of PE improved memory and learning in animal and human models. Zhang et al. (2019) demonstrated that a four-month treadmill protocol was able to prevent the decline in spatial memory capacity. When parents performed exercise, there was an improvement in offspring cognition (Park and Kim [32]), demonstrating the characteristics related to the active phenotype (Pirola and Leandro [63]). In humans, exercise has been shown to improve learning and memory through structural and neurochemical changes in the hippocampus (Hötting and Röder [64]). Moreover, a growing literature suggests that exercise, specifically aerobic exercise, may attenuate cognitive impairment and reduce dementia risk (Gobinath et al. [65]).

Aerobic exercise also resulted in decreased anxiety and depression in animal models, even in a stressful situation (administration of corticosterone). PE has an action similar to fluoxetine (selective serotonin reuptake inhibitor) on the treatment of depression and anxiety (Vahid-Ansari and Albert [66]). Thus, like the action of fluoxetine on neurogenesis, the practice of PE increases the plasticity of the hippocampus, promoting changes on serotonin metabolism and synaptic plasticity (Micheli et al. [67]). As well, both PE and fluoxetine are effective in combating depression and anxiety caused by stress or neurodegenerative diseases (Harvey et al. [68]).

These results demonstrated that PE is associated with improved physical health, satisfaction with life, cognitive functioning, and psychological well-being (Aguiar et al. [26]; Gobinath et al. [65]; Kadariya and Gautam [69]). However, it is not yet known how the variables linked to physical training (intensity, volume, and frequency) can modulate neuroplasticity and its mechanisms.

## 5. Conclusion

PE was able to promote neuroplasticity through increasing cell signaling and high neuronal growth and differentiation in animal models. In humans, neuroplasticity was observed by increasing the white and gray matter in various brain areas after different PE protocols. Additionally, PE also promoted improvements in cognitive function, such as learning and memory.

## Figures and Tables

**Table 1:** Description of included animal studies.

Author (year)	Aim	Animal species/experimental groups	Brain tissues	Analysis	Protocol of physical exercise
Gomes da Silva et al. [23]	To investigate the morphological and functional hippocampal changes in adult rats submitted to daily treadmill exercise during the adolescent period.	Male Wistar rats at 21 days of age were divided into two groups: exercise (*n* = 27) and control (*n* = 27).	Hippocampus	Immunofluorescence; ELISA; immunoblotting	An aerobic physical exercise protocol (running on a treadmill) was performed. Only animals that were classified as medium, above average, and good runner were included in the procedure. The physical exercise protocol was performed between days 21 and 60 of life. Each session started with a 5-minute warm-up at 8-10 m/minute. The running time and speed were gradually increased, reaching a maximum of 18 m/min for 60 min. Animals in the control group were treated in the same way as animals in the exercise group.

Aguiar et al. [24]	Investigate whether long-term light physical exercise on a running wheel or treadmill improves spatial learning, memory, and plasticity of the hippocampus in elderly rats.	Female Wistar rats aged 24 months were divided into two groups: exercised (*n* = 17 animals) and sedentary controls (*n* = 18 animals).	Hippocampus	Western blotting; RT-PCR	Animals underwent an adaptation period for 1 week on a treadmill, with a daily session of 3 minutes at a speed of 2 m/min. Then, the aerobic exercise protocol was started, which was performed for 4 consecutive weeks, with 4 sessions per week. Each exercise session consisted of a warm-up session (3 min, 2 m/min), followed by two sessions of running sessions (4-6 min) at a constant belt speed 10 m/min. Each session was spaced by a 1-minute rest interval.

Aguiar et al. [25]	To analyze the effects of physical exercise on mitochondrial physiology, anxiodepressive-like behaviors and neuroplasticity in mice.	Male C57BL/6 mice aged 8-10 weeks old were divided into two groups: voluntary exercise (*n* = 16) and control group (*n* = 16).	Hippocampus	RT-PCR; HPLC; Western blotting; spectrophotometrical assays for assessing	A voluntary physical exercise protocol was carried out in individual cages equipped with a steering wheel to stimulate voluntary exercise for 6 weeks (Aguiar et al. [26]).

Bhattacharya et al. [27]	To determine the effects of EGCG (∼250 mg/kg/day), B-ALA (∼550 mg/kg/day), and their combination with voluntary wheel running exercise.	91 male BALB/cJ mice at 10 weeks of age were divided into eight groups: control (*n* = 11 sedentary and 11 runners), B-ALA (*n* = 11 sedentary and 12 runners), EGCG (*n* = 12 sedentary and 11 runners), or EGCG and B-ALA combined (*n* = 12 sedentary and *n* = 11 runners).	Hippocampus	Immunohistochemistry	A voluntary physical exercise protocol was carried out for 39 days. Wheel rotations were monitored continuously in one-hour increments throughout the experiment via magnetic switches interfaced to the computer.

Brockett et al. [28]	To investigate whether running alters performance on cognitive tasks that require the prefrontal cortex and whether any such changes are associated with astrocytic, as well as neuronal plasticity.	Adult male Sprague-Dawley male rats are divided into two groups: sedentary controls (*n* = 18) and runners (*n* = 18) groups.	Hippocampus; medial prefrontal cortex; orbitofrontal cortex	Immunolabeling for astrocyte and synaptic markers; DiI impregnation	A protocol of voluntary physical exercise in a running wheel was performed for 12 days. The running distance was recorded daily digital counters mounted on the racing wheels.

Kim et al. [29]	To investigate the effect of treadmill exercise on impairment of cognitive function in relation with hippocampal neuroplasticity using high-fat diet-induced obese mice.	C57BL/6 male mice at four weeks of age were divided into four groups: control group (*n* = 10), control and exercise group (*n* = 10), high-fat diet group (*n* = 10), and high-fat group and exercise (*n* = 10).	Hippocampus	Western blotting; immunohistochemistry	An aerobic exercise protocol was performed in treadmill for 20 weeks.

Vilela et al. [30]	To investigate the effect of aerobic and strength training on spatial memory and hippocampal plasticity in aging rats.	Male Wistar rats of 24 months old were divided into three groups: (*n* = 6/group) untrained, aerobic training, and strength training groups.	Hippocampus	Western blotting	Two physical exercise protocols were performed. The aerobic exercise protocol was performed on a running treadmill. Each session lasted 50 minutes and there was an interval of 48 hours between sessions. The anaerobic exercise protocol was performed through strength training. Climbing with 1 m inclined at 85° was carried out with weight attached to the tail. The weight attached to the tail was gradually increased from 50 to 100% during the 8 weeks of training. Three to five sets of 8 to 12 repetitions, with a 1-minute rest between repetitions and a 2-minute rest between sets, were performed for 3 or 4 days/week. Each session lasted 40 to 50 minutes, with an interval of 48 hours between sessions.

de Senna et al. [31]	To investigate the effects of physical exercise to prevent or reverse spatial memory deficits produced by diabetes and some biochemical and immunohistochemical changes in hippocampal astrocytes of type 1 diabetes mellitus model.	Three-month-old male Wistar rats were divided into four groups: nontrained control (*n* = 15), trained control (*n* = 15), nontrained diabetic (*n* = 13), and trained diabetic (*n* = 13).	Hippocampus	Immunohistochemistry; morphological analysis of astrocytes.	The aerobic physical exercise protocol was performed on a running treadmill at moderate intensity. The protocol took place once a day for 5x a week for 5 weeks.

Park and Kim [32]	To assess the effects of paternal physical exercise on spatial learning ability in relation with hippocampal neuroplasticity in the rat pups born from the obese maternal rats.	Male and female Sprague-Dawley rats of 4 weeks old were divided into four groups: nonexercising male and normal female group (*n* = 5), exercising male and normal female group (*n* = 5), nonexercise male and obese female group (*n* = 5), and exercise male and obese female group (*n* = 5).	Hippocampus	Immunohistochemistry; Western blotting	An aerobic exercise protocol was performed on a running treadmill. The exercise was performed once a day and 6 days a week for 12 consecutive weeks [first 3 weeks (30 minutes—speed: 10 min/min); 4-6 weeks (40 minutes—10 m/min); 7-9 weeks (30 minutes—15 m/min); 10 to 12 weeks (40 min—15 m/min)].

Park et al. [33]	To determine whether symptoms of chemo brain and disruptions in the neuroplasticity and functioning of hippocampal mitochondria can be prevented or relieved by exercise.	Male Wistar rats of 6 weeks olds were divided into four groups: control (*n* = 15), control and exercise (*n* = 15), DOX-induced chemo brain (*n* = 15), and DOX-induced chemo brain and exercise (*n* = 15).	Hippocampus	Immunofluorescence; immunohistochemistry; Western blotting	An aerobic exercise protocol was performed on a running treadmill. The exercise was performed once a day (30 min at 10 m/min) and six days per week for 4 consecutive weeks.

Park et al. [34]	To investigate whether the decline in cognitive function caused by a high-fat diet could be improved through exercise by examining insulin signaling pathways and neuroplasticity in the hippocampus.	Male C57BL/6 mice of 4 weeks old were divided into five groups: control (*n* = 20), control and exercise (*n* = 20), exercise (*n* = 20), high-fat diet (*n* = 20), and high-fat diet and exercise (*n* = 20).	Hippocampus	Immunofluorescence; immunohistochemistry; Western blotting	An aerobic exercise protocol was performed on a running treadmill. The treadmill exercise started 20 weeks after taking the HFD. The exercise was performed once a day and six days per week for 12 consecutive weeks [1-2 weeks (30 min—10 m/min); 3-4 weeks (40 min—10 m/min); 5-6 weeks (30 min—13 m/min); 7-8 weeks (40 min—16 m/min); 9-10 weeks (40 min—16 m/min); 10-12 weeks (50 min—16 m/min)].

Yau et al. [35]	Examine the effects of training on the administration of corticosterone on the hippocampus neurogenesis, cell proliferation and differentiation, synaptic protein expression, expression of neurotrophic factors, and behavioral analysis.	Adult male Sprague-Dawley rats were divided into four groups (*n* = 4‐6): (1) control rats (CON); (2) CORT- treated rats that could run only during the 2-week CORT administration period (CR); (3) CORT-treated rats with the 2 weeks prior running only (PR); and (4) CORT-treated rats that could run both prior and concurrently with the CORT administration period (PR + CR).	Hippocampus	Immunohistochemistry; Western blotting	A protocol of voluntary physical exercise in a running wheel was performed for 34 days. The wheels were then unlocked for the rats with running for 14 days prior to the 50 mg/kg corticosterone treatment.

Pan et al. [36]	To explore the specific role of physical exercise in novel object recognition memory after stroke and the exact cortical regions in which memory is restored by physical exercise.	Spontaneously hypertensive rats of 10-12 weeks old were divided into four groups (*n* = 20 rats/group): control groups; tMCAO (2 d) group, in which rats underwent tMCAO surgery and NOR tests were performed 2 days later; the tMCAO (28 d) group, in which NOR tests were performed at 28 days post-tMCAO; and a tMCAO (28 d)+PE group, in which tMCAO was established and rats exercised in a running wheel for 26 consecutive days starting at the third day post-tMCAO.	Entorhinal cortex	Histochemistry; Western blotting	The physical exercise protocol was performed on a motorized racing wheel for 26 days from day 3 after treatment with tMCAO. During the first 12 days, the speed of 3 m/min was maintained for 20 minutes, twice a day. In the following 14 days, the speed was maintained at 6 m/min.

Rabelo et al. [37]	To evaluate whether the intrinsic capacity for physical exercise influences dopamine neuroplasticity induced by physical training.	Male Wistar rats of 2 months old were divided into three groups: low performance LP (*n* = 8), standard performance (SP) (*n* = 8), and high performance (HP) rats (*n* = 8) which were randomized into the SED and TR groups.	Not informed	PCR	The aerobic exercise protocol was performed on a treadmill for 6 weeks, 5 times a week. The training time was obtained through an exercise test (40% of the maximum time obtained during the test of 60% *V*_max_) and the speed was kept constant. The duration of the training sessions was increased by 10% per week (except for the transition from the 3rd to the 4th week).

Seo et al. [38]	To evaluate whether exercise can improve psychiatric status and cognitive functioning, increasing the mitochondrial function of the hippocampus and neuroplasticity in a model of rats with posttraumatic stress disorder.	Male Sprague-Dawley of 4 weeks old were divided into four groups: control (CON) group, a control and exercise (CON+EX) group, a PTSD group, and a PTSD and exercise (PTSD+EX) group (*n* = 15 in each group).	Hippocampus	Immunofluorescence; Western blotting; mitochondrial analysis	The aerobic exercise protocol was performed on a treadmill once a day, 6 days a week, for 4 consecutive weeks. In the first two weeks, the exercise lasted 30 min with a speed of 10 m/min. In the third week, the rats performed 40 minutes of exercise at a rate of 12 m/min. In the fourth week, the exercise lasted 50 minutes at 13 m/min.

B-ALA: *β*-alanine; CORT: corticosterone; Dox: doxorubicin; ELISA: enzyme-linked immunosorbent assay; ECGC: epigallocatechin-3-gallate; EX: exercise; GFAP: glial fibrillary acidic protein; HPLC: high-performance liquid chromatography; NOR: novel object recognition; PE: physical exercise; PTSD: posttraumatic stress disorder; RT-PCR: real-time polymerase chain reaction; SED: sedentary; tMCAO: transient middle cerebral artery occlusion; TR: trained.

**Table 2:** Description of the included studies with human.

Author (year)	Aim	Sample description	Brain tissue	Other analysis	Study design/protocol of physical exercise
Eftekhari and Etemadifar [39]	To determine the chronic effect of Mat Pilates on serum levels of interleukin-10 and brain-derived neurotrophic factor in women with multiple sclerosis.	25 women are suffering from relapsing-remitting multiple sclerosis with expanded disability status scale 2-6, based on McDonalds criteria. They were divided into two groups: control group (*n* = 12; age (years) = 34.46 ± 7.29) and Pilates training group (PT) (*n* = 13; age (years) = 31.41 ± 8.89).	—	ELISA	This is a randomized controlled study, which was carried out from April 2015 to June 2015. The anaerobic exercise protocol was performed through Mat Pilates training and lasted eight weeks, three times a week, lasting 30-40 min/day. The exercises were performed at low to moderate intensity according to the patient's performance. The intensity of the exercise gradually increased with the inclusion of more repetitions (3-10), decreasing the rest time and increasing the number of sets (1-2). Blood collection was carried out between 8 and 9 am to determine the serum levels of pre- and posttest. The posttest blood sample was collected 48 hours after the last Pilates session.

Müller et al. [40]	To assess whether a dance training program that emphasizes constant learning of new movement patterns is superior in terms of neuroplasticity to conventional conditioning with repetitive exercise and whether the extension of the training duration has additional benefits.	62 healthy (63 to 80 years old) were recruited through advertisements in local newspapers. The following exclusion criteria were adopted: claustrophobia, tinnitus, metal implants, tattoos, diabetes mellitus, depression, cognitive deficits, neurological diagnosis, and regular physical exercise (1 h/week). 10 participants were excluded from the criteria. Only 22 participants completed the entire intervention. These were divided into two groups: dancing group (*n* = 12) and sport group (*n* = 10).	Gyrus and right parahippocampal	ELISA; MRI	This is a controlled intervention study with a total duration of 18 months. The intervention was carried out in two periods: first period (twice a week in sessions of 90 minutes for 6 months) and second period (training once a week in sessions of 90 minutes for 12 months). Two protocols were used: dance group (participants were asked to learn new sequences of movement, which required coordination; each rhythm was changed after the fourth session). Sport group (participants performed strength-resistance training, with low demand for coordination; each session lasted 20 minutes). The two training programs were comparable in terms of intensity, duration, and frequency. Baseline assessments were made after 6 and 18 months of training.

Ji et al. [41]	To assess whether motor skill causes changes in plasticity after exercise in different modalities.	24 healthy individuals (70 ± 7.78 years old; 12 women) were recruited through advertisements. These were divided into two groups: control (*n* = 12; 7 women; 73 ± 8 years old) and trained group (*n* = 12; 5 women; 67 ± 6.4 years old).	Dorsolateral prefrontal cortex, posterior cingulate, precuneus cortex, hand motor area, occipital lobe, and cerebellum	MRI	The study was a quasiexperiment. A physical exercise protocol was carried out that had four domains (aerobic, balance, weight lifting, and yoga). Participants were instructed to practice at home for 30 min every day for 6 weeks.

Rehfeld et al. [42]	To evaluate the effects of a dance program in the elderly on brain plasticity.	62 volunteers were selected through a local advertisement. After the exclusion criteria, 52 elderly people (25 men; 27 women) aged 63 ± 80 years were randomly assigned to the experimental dance group (DG) and the control group to the sports group (SG). In the end, 38 participants completed the intervention. Dance group (*n* = 20) and sport group (*n* = 18).	Frontal, temporal, cortical, and cerebellar regions	ELISA; MRI	This is an intervention study that used two protocols: dance intervention (subjects were trained to accurately memorize and access different rhythms and step sequences in space, all under accuracy and time pressure) and sport intervention (each session included three different units: endurance training, strength-endurance training, and flexibility training). The protocols happened twice a week for 90 minutes and six months long.

Woost et al. [43]	To investigate whether a sequential combination of physical and spatial training in young, healthy adults elicits an additive effect on training and transfer gains.	99 volunteers aged between 18 and 35 participated in the study.	Hippocampus	ELISA; MRI	The study presents an experimental design. Participants performed eight sessions: (1) three weeks of 20 minutes of cycling per day classified based on high-intensity training between T0 and T1 (ERGO); (2) five weeks with 16 sessions of 30 minutes of space training between T1 and T2 (MAZE); and (3) a sequential combination of both (COMBO) or rested as passive controls.

Rogge et al. [44]	To test if balance training, challenging the sensory-motor system and vestibular self-motion perception, induces structural plasticity.	Participants were recruited through public announcements. Healthy adults between 19 and 65 years of age who did not report regular exercise were eligible for the study. 59 participants were randomized between groups; however, only 38 completed the study (24 women/14 men). Participants were divided into two groups: balance group (*n* = 19) and relaxation group (*n* = 18).	Hippocampus; basal ganglia	MRI	It is an intervention study, where the participants carry out 12 training sessions, with two sessions per week, each lasting 50 min. Two protocols were performed: balance training (eight different stations per session) and relaxation training (progressive muscle relaxation and autogenic training).

ELISA: enzyme-linked immunosorbent assay; MRI: magnetic resonance.

**Table 3:** Description of the main results of animal studies.

Author (year)	Neuroplasticity outcomes			Functional outcomes
	Neurotrophins and receptors	Cell signaling	Cell growth and differentiation	
Gomes da Silva et al. [23]	An increase in BDNF expression was observed in the hippocampus (*p* = 0.00) and TrkB (*p* = 0.03) in the exercise group when compared to the control group. There were no differences for P75NRT receptor (*p* = 0.69).	**—**	No significant differences in neuronal density were found between the exercise group and control groups [CA1 (*p* = 0.49); CA3 (*p* = 0.66); DG (*p* = 0.69)]. However, the density of mossy fibers was higher in the hippocampal formation of the exercise group (*p* = 0.01) when compared to the control group.	Learning and memory of animals from exercise and control groups were analyzed in the water maze for five days. Latency (*p* = 0.00) and length of the swimming path (*p* = 0.00) in the exercise group were significantly shorter than in the control group. However, there were no differences in speed (*p* = 0.65), showing that the results were not influenced by physical conditioning. On day 66, the platform was removed from the water maze to assess the time spent in each of the four imaginary quadrants. The results showed that both groups showed preferences for the previous quadrant (*p* < 0.05). On day 96, the platform was replaced in the water maze to analyze long-term memory. It was observed that the latency in finding the platform was lower in the exercise group (*p* = 0.00) compared to the control group.

Aguiar et al. [24]	BDNF mRNA expression (*p* = 0.05) and BDNF protein levels (*p* = 0.05) were higher in the hippocampus of elderly rats after short light intensity exercises.	Phosphorylation of Akt (*p* = 0.05) and CREB (*p* = 0.05) was greater in the hippocampus of trained elderly rats when compared to the control.	**—**	Learning and memory were analyzed in the water maze. A reduction in the escape latency of the exercised group was observed when compared to the control group (*p* = 0.05). In addition, more time was spent in the correct quadrant (*p* = 0.05) in the exercise vs. control group. There were no differences in swimming speed (*p* = 0.48) and distance covered (*p* = 0.64).

Aguiar et al. [25]	Physical exercise increased the expression of BDNF (*p* < 0.05) and GDNF (*p* < 0.05) in the hippocampus.	Physical exercise increased the phosphorylation of the element responsive to cAMP and CREB protein in the hippocampus (*p* < 0.05).	**—**	The effects of physical exercise on depression-like and anxiety-like behaviors were evaluated. In the elevated plus maze (EPM) test, it was observed that PE promoted an anxiolytic effect indicated by increased open arms by exercised rats compared to the control group [EPM open arms: % total arms entries (*p* < 0.05); time of investigation (*p* < 0.05)]. In the open field, there was an increase in the exploration of the central regions of the open field in exercised animals. The increase in locomotion (*p* < 0.05) and exploration time in the center of the open field (*p* < 0.05) was greater in the trained vs. sedentary group. In the tail suspension test, the exercised group showed reduced immobility (*p* < 0.05). However, no differences were found between groups regarding latency time (*p* = 0.53).

Bhattacharya et al. [27]	—	—	Exercise increased the total number of BrdU+ cells in the granular layer of the dentate gyrus compared to the sedentary group (*p* < 0.0001).	To assess the conditioning of contextual fear, physical exercise increased the duration of freezing in the original context (*p* < 0.00). In the assessment of fear conditioning, physical exercise promoted a longer duration of freezing behavior (*p* = 0.00).

Brockett et al. [28]	Runner animals showed an increase in PSD-95 in the hippocampus (*p* = 0.01), medial prefrontal cortex (*p* = 0.00), orbitofrontal cortex (*p* = 0.00), and perirenal cortex (*p* = 0.02).	—	An increase in body area of astrocyte cells was observed in the hippocampus (*p* = 0.02), medial prefrontal cortex (*p* = 0.03), and orbitofrontal cortex (*p* = 0.00) in the runners group when compared to the sedentary group. No differences were observed in the perirenal cortex. Concerning the density of the dendritic column, runner animals had an increase in the apical (*p* = 0.0001) and basal dendrites of the 2/3-layer pyramidal neurons (*p* = 0.0001) in the medial prefrontal cortex. There was also a significant increase in apical (*p* = 0.001) and basal (*p* = 0.00004) trees in runner animals.	Object memory testing was used to evaluate memory. Runners showed the highest discrimination ratio on the object in place task (*p* = 0.00). On the other hand, there was no difference in discrimination rates between groups on the novel object preference after 12 days of running (*p* = 0.4). To assess cognition, an attentional set-shifting task was performed. Runners showed an improvement in simple (*p* = 0.01), reversal (*p* = 0.00), and extradimensional (*p* = 0.01) discrimination, changing the attention-changing task in terms of the number of attempts to reach the criterion. However, sedentary mice also performed the task as expected.

Kim et al. [29]	Physical exercise increased the expression of BDNF and TrkB compared to the control and high-fat group (*p* < 0.05).	—	Treadmill exercise increased the number of DCX-positive and BRDU-positive cells in control and high-fat diet-induced obese mice (*p* < 0.05).	Memory was assessed by the Y-maze test. Treadmill exercise alleviated deterioration of spatial and short-term memory in control mice [spontaneous alternation (*p* < 0.05), correct number (*p* < 0.05), and error number (*p* < 0.05)] and obese patients induced by a high-fat diet.

Vilela et al. [30]	Aerobic exercise showed an increase in PSD-95 levels when compared to the other groups (*p* < 0.05). However, the two physical exercise protocols showed improvements over the levels of pNMDA (*p* < 0.05), BNDF (*p* < 0.05), and p75NTR (*p* < 0.05) when compared to the control. TrkB levels were decreased after aerobic exercise (*p* < 0.05).	CREB increased after training for both exercise protocols (*p* < 0.05).		The Barnes maze test was used to assess memory and cognition. The latency to find the escape orifice was reduced and spatial memory (increase in the time spent in the destination quadrant) was improved after the strength and aerobic exercise (*p* < 0.05) compared to the sedentary group.

de Senna et al. [31]	—	—	A significant increase in the number of astrocytic ramification in all directions was observed in trained diabetic rats when compared to the diabetic group (*p* < 0.05).	Memory was assessed using the place recognition test. After physical training, the analysis of the exploration time of trained diabetic rats was greater than in the diabetic group (*p* < 0.05).

Park and Kim [32]	Paternal exercise enhanced BDNF (*p* < 0.05) and TrkB (*p* < 0.05) expressions in the male rat pups from the obese maternal rats.	—	Paternal exercise increased cell differentiation (DCX-positive cells; *p* < 0.05) and cell proliferation (BrdU-positive cells; *p* < 0.05) in the hippocampus of male rat pups from the obese maternal rats.	Spatial learning ability was evaluated using the Morris water maze task. Paternal exercise reduced the escape latency (*p* < 0.05) and time in probe quadrant (*p* < 0.05), improving the spatial learning capacity in male offspring of obese rats.

Park et al. [33]	Physical exercise reduced the effects of CHEMO, increasing the levels of BDNF (*p* < 0.05) and TrkB (*p* < 0.05).	—	Exercise increased levels of cell proliferation in animals that received CHEMO [BRDU/Neun-positive cells in dentate gyrus (*p* < 0.05)].	Short-term memory was assessed using the step-down avoidance task, and Morris water maze task was performed to assess spatial learning and working memory. A reduction in the step-down latency time (*p* < 0.05), escape latency (*p* < 0.05), and longer time in the quadrant probe (*p* < 0.05) was observed in CHEMO+EX compared to the CHEMO group.

Park et al. [34]	Exercise reduced the effects of the high-fat diet, increasing the hippocampal levels of BDNF (*p* < 0.05) and TrkB (*p* < 0.05).		Exercise reduced the effects of the high-fat diet, increasing cell proliferation [number of Brdu/NeuN-positive cells in the dentate gyrus (*p* < 0.05)] and cell differentiation [number of DCX-positive cells in the dentate gyrus (*p* < 0.05)].	The Morris water maze test was used to assess spatial memory. Exercise reduced the effects of the high-fat diet, reduced escape latency (*p* < 0.05), stepped down latency time (*p* < 0.05), and increased time in probe quadrant (*p* < 0.05).

Yau et al. [35]	Treadmill exercise improved levels of PSD-95 (*p* < 0.05) and SYN (*p* < 0.05) reduced by treatment with costicosterone.	Treadmill exercise improved IGF-1 levels (*p* < 0.05) reduced by treatment with costicosterone.	Treadmill exercise improved levels of CIdU-positive cells (*p* < 0.05), number of proliferating cells in dentate gyrus (*p* < 0.05), CIdU-positive cells in the dentate gyrus (*p* < 0.05), IdU-labeled cells in dentate gyrys (*p* < 0.05), BrdU-positive cells (*p* < 0.05) and increased neuronal differentiation (DCX cells, *p* < 0.05) reduced by treatment with costicosterone.	Depression-like behavior was measured according to the method of the forced swim test. Continuous running decreased immobility time (*p* < 0.05) and decreased depression-like phenotypes compared to the CORT group.

Pan et al. [36]	Physical exercise increased the reduced levels of SYN (*p* < 0.001) and PSD-95 (*p* < 0.001) in the entorhinal cortex in tMCAO rats.	—	Physical exercise improved cell proliferation [nestin-positive cells (*p* < 0.01), Ki-67-positive cells (*p* < 0.001), and TUNEL-positive cells (*p* < 0.001)] in transient rats with middle cerebral artery occlusion (tMCAO).	Memory was assessed by the novel object recognition test. Physical exercise improved memory (discrimination ratios, *p* < 0.01) of the recognition of new objects in transient mice with occlusion of the middle cerebral artery (tMCAO).

Rabelo et al. [37]	Physical training elicited an increase in Gdnf mRNA levels in the CPu of low performance rats (*p* < 0.01), whereas it decreased Bdnf expression only in high-performance rats (*p* < 0.05).	—	—	—

Seo et al. [38]	There was an increase in hippocampal expression of BDNF (*p* < 0.05) in animals trained with posttraumatic disorder. However, there was no difference in TrkB levels.	—	There was no difference on positive cells for BrDU/NeuN and DCX in the dentate gyrus between the groups, with no differences on neurogenesis and cell differentiation.	The open field, elevated plus maze, forced swimming test, and Morris water test were performed to assess depressive-like behavior, anxiety, depressive state, and spatial learning and working memory, respectively. The exercise group showed an increase in open arms (*p* < 0.05) and time in open arms (*p* < 0.05) when compared to the PTSD group. There was a reduction in immobility time (*p* < 0) and average distance in the centers (*p* < 0) in the PSTS+EX group. Thus, a reduction in anxiety, depression-like behavior, and a depressed state was observed in the trained animals. There was no effect of exercise on escape latency and time in the probe quadrant, and average distance from the center demonstrating no improvement in memory and learning.

AKT: protein kinase B; APE1: apurinic/apyrimidinic endonuclease 1; BNDF: brain-derived neurotrophic factor; CAMP: cyclic adenosine 3′,5′-monophosphate; CREB: cyclic amp-response element binding protein; CR: CORT-treated rats that were allowed to run only during 2 weeks; CORT: corticosterone; CPu: caudate-putamen; DAT: dopamine transporter; DOPAC: 3,4-dihydroxyphenylacetic acid; DA: dopamine; DCX: doublecortin; GDNF: glial cell-derived neurotrophic factor; HP: high performance; LP: low performance; mRNA: messenger RNA; PDS-95: postsynaptic density protein 95; p75NTR: p75 neurotrophin receptor; PR: CORT-treated rats of 2 weeks prior to running only; PE: physical exercise; TrkB: tropomyosin receptor kinase B; TR: trained; tMCAO: transient middle cerebral artery occlusion; 5-HT: serotonin.

**Table 4:** Description of the main results of human studies.

Author (year)	Neuroplasticity outcomes		Cognitive outcomes
	Neurotrophic factors	Structural changes	
Eftekhari and Etemadifar [39]	An increase in BDNF levels was observed after an 8-week Pilates training (*p* = 0.03).	**—**	**—**

Müller et al. [40]	Dancing group presented an increase in BDNF level (*p* < 0.05); after 6 months, there were no differences in the sport groups.	Through MRI, an increase in gray matter was observed on the left side of the precentral gyrus in the dance group after 6 months (*p* < 0.05). At 18 months, there was an increase in gray matter in the right *parahippocampal* gyrus in the dance group (*p* < 0.05).	Neuropsychological tests were performed to assess short- and long-term memory (verbal learning test and attentional performance test). Both groups demonstrated improvements in short-term memory (early VLMT recovery, *p* = 0.00) and long-term (long-term free verbal recall, *p* = 0.04; long-term verbal recognition, *p* = 0.00) after 6 and 18 months of training in both tests. However, there was no difference between groups.

Ji et al. [41]	—	The trained group showed an increase in gray matter in the dorsolateral prefrontal cortex, posterior cingulate/precuneus cortex, hand motor area, occipital lobe, and cerebellum. There was no difference in the striatum.	The following tests were used to assess neuropsychological function: immediate, delayed, and recognition recall from Hopkins Verbal Learning Test-Revised (HVLT_Imm, HVLT_Delay, and HVLT_Recog); immediate and delayed story recall from the rivermead behavioral memory test (RM_Imm and RM_Delay), WAIS-III digit symbol substitution modality test (DSST), WAIS-III digit span, trail making test (trails A and trails B), and Stroop color and word test. The physical training group performed the cognitive tests before and after the 6-week training. All neuropsychological test scores increased after exercise, except HVLT_Recognition. An improvement was observed on memory (*p* = 0.01) and executive function (*p* = 0.01). The physical training group showed significantly greater improvement in the discrimination rate than the control group (*p* = 0.03) after 6 weeks.

Rehfeld et al. [42]	The dance group showed an increase in plasma BDNF levels (*p* < 0.018) when compared to the sport group.	After the intervention, the dance group showed greater volume in gray matter in frontal and temporal cortical areas, including the anterior cingulate and medial cortex, the left supplementary motor area, the left precentral gyrus, the left medial frontal gyrus, the left insula, the upper left temporal gyrus, and the left post-entral gyrus compared to the sport group. However, there was greater volume in the occipital and cerebellar regions in the sport group. In white mass, there was greater volume in the dance group in the truncus and splenium of the corpus callosum, in the right and left frontal and right parietal. In the sport group, there was a greater volume of white matter in the right temporal and right occipital.	Tests were used to assess cognition (alertness, go/no go, divided attention, and flexibility), processing speed (trail test), verbal fluency of words, short-term memory and working memory (digit span forward and backward of the Wechsler Memory Scale), verbal episodic memory (verbal learning and memory task), and visuospatial memory (Rey-Osterrieth complex figure test). The visuospatial memory improved after the two training sessions (*p* < 0.00) as well as for late recovery (*p* < 0.001). The other analyses did not obtain differences between the beginning and the end of the protocol. There were no differences between groups.

Woost et al. [43]	BDNF levels did not differ over time (*p* = 0.09).	—	Cognitive performance was assessed using the following tests: spatial cognition assessment (FRS), “Dresden Spatial Navigation Task,” human analogue of the “Morris water maze” (huWMZ), “location memory” subtest of the “Berlin Intelligence Structure Test” (BIS) 51 and memory evaluation: VVM, subtests through “figures,” “data,” and “matrices” of “Intelligence Structure Test 2000R” (IST), and “California Verbal Learning Test” (CVLT). The groups did not differ on the longitudinal change in cognitive performance (*p* > 0.05).

Rogge et al. [44]	—	There were no differences between subcortical gray matter volume in the hippocampus of either group, in the right hemisphere (*p* = 0.226) or in the left (*p* = 0.743). The balance group showed a reduction in putamen gray matter volume bilaterally compared to the relaxation group (left hemisphere, *p* = 0.019; right, *p* = 0.025). In addition, better balance performance caused greater precentral cortical thickness (left hemisphere, *p* = 0.002; right, *p* = 0.426). Participants who showed the greatest increase in balance performance showed a greater decrease in gray matter volume in the left putamen (*p* = 0.002).	—

BDNF: brain-derived neurotrophic factor; HTLV: recognition recall from Hopkins Verbal Learning Test-Revised; IGF-1: insulin-like growth factor-1; VEGF: vascular endothelial growth factor.
